# Health-related quality of life and its association with socioeconomic status and diet diversity in Chinese older adults

**DOI:** 10.3389/fpubh.2022.999178

**Published:** 2023-01-20

**Authors:** Chichen Zhang, Jiachi Zhang, Shujuan Xiao, Lei Shi, Yaqing Xue, Xiao Zheng, Xue Benli, Yiming Chen, Xinru Li, Yan Kai, Yuxi Liu, Guangqing Zhou

**Affiliations:** ^1^Department of Health Management, Nanfang Hospital, Southern Medical University, Guangzhou, China; ^2^School of Health Management, Southern Medical University, Guangzhou, China; ^3^Institute of Health Management, Southern Medical University, Guangzhou, China; ^4^School of Public Health, Southern Medical University, Guangzhou, China; ^5^Shunde Hospital, Southern Medical University, Guangzhou, China; ^6^School of Humanities and Management, Institute for Health Law and Policy, Guangdong Medical University, Dongguan, China

**Keywords:** health-related quality of life (HRQoL), diet diversity, socioeconomic status, older adults, health management

## Abstract

**Objectives:**

The study aimed at examining the combined association of socioeconomic status (SES) and diet diversity (DD) with health-related quality of life (HRQoL) and exploring whether DD played a mediating role in the relationship between varied SES and HRQoL among Chinese older persons.

**Method:**

A multi-stage random sampling method was conducted in Shanxi Province of China, with 3,250 older adults participating in this cross-sectional survey. SES was divided into groups by quartiles and DD by means, and these variable groups were combined in pairs to generate a total of eight combinations. The PROCESS macro developed by Hayes was employed for the simple mediation analysis.

**Results:**

Compared with the reference group (those with both high SES and high DD), older adults who were classified to have lower SES or DD had elevated odds of having worse HRQoL: low SES/ low DD (*OR* = 1.65, 95% CI 1.41–2.92); low SES/ high DD (*OR* = 1.45, 95% CI 1.17–1.80); middle low SES/ low DD (*OR* = 1.43, 95% CI 1.24–1.65); middle low SES/ high DD (*OR* = 1.23, 95% CI 1.03–1.47); upper high SES/ low DD (*OR* = 1.41, 95% CI 1.21–1.65); and high SES/ low DD (*OR* = 1.30, 95%CI 1.10–1.53). The mediation analysis revealed that DD mediated the relationship between SES and HRQoL (*B*=0.011, 95% CI 0.008–0.013), with its indirect effects accounting for 39.29% of the total effects.

**Conclusions:**

These findings highlighted the role of DD as a mediator of the relationship between SES and HRQoL. As DD could be protective, modifiable, and easy for older adults to understand and implement, village clinics and community health stations should work collaboratively to design proper DD intervention measures for better HRQoL.

## Introduction

With increasing average life expectancy, populations are now living longer but longevity does not necessarily mean that people have a better life quality (1). Health-related quality of life (HRQoL) is a multi-dimensional concept capturing the overall health and wellbeing of individuals or groups with various socio-economic characteristics (2). Among the many determinants of older adults' HRQoL, the researchers have paid close attention to socioeconomic status (SES), a measure of people's combined economic and social status representing the individuals' access to culturally relevant resources needed to succeed in society (3, 4). The “health choice theory” and the “social causation theory,” two theoretical explanations for the relationship between SES and HRQoL, both recognized the positive relationship between SES and health. The relationship between SES and health in our study may be more consistent with the social causation theory given the observation time point and the suggestion made by a prior Chinese cohort study that older individuals' SES had been formed before their retirement (5, 6). Low SES was associated with poor HRQoL in China (7), with a similar trend also observed in other countries such as Korea and Greek (8, 9). Although the relationship between SES and HRQoL has been well established, how the mechanism works particularly remained less apparent.

People's behaviors and observable attributes could be affected by SES, and these behaviors could also insert an impact on people's health (10). As a major component of lifestyle behaviors, dietary behavior was one of the key modifiable factors in promoting health and longevity (11). A local study found that unhealthy dietary behavior was highly prevalent and was associated with older adults' HRQoL in China (12). Therefore, we have begun to consider the role of dietary behaviors that might help to explain the relationship between SES and HRQoL. Dietary diversity (DD) is an indicator providing an overall assessment of dietary behaviors (13), reflecting people's nutrient adequacy and diet quality (14, 15). The importance and value of DD have been widely recognized by both the national and international dietary guidelines (16, 17). Previous population-based studies have clarified the relationship between SES and DD. In eight Latin American countries, Gomez et al. reported that respondents' DD increased with SES (18). Similar associations had also been observed among adults from Mexico (19), rural Mali (20), and other low and middle-income countries (21). These findings supported the hypothesis that the SES may subsequently beget people's DD.

In terms of the association between DD and HRQoL, it has also been confirmed by previous research. According to a 20-year longitudinal study of 91,993 Chinese citizens over the age of 65, poor DD was significantly associated with a poor quality of life after being adjusted for potential confounders (22). Other cross-sectional and longitudinal studies have also identified that DD could benefit older adults' physical functional capacity (23) and memory status (24), as well as suppress cognitive impairment (25) and even mortality (26). Additionally, it was shown that DD could help older Chinese populations to achieve a better aging process (27). Taken together, the pathway through DD may be one of the intermediate mechanisms between SES and HRQoL among older adults, and it is necessary to elucidate the potential mediating role of DD on the association among Chinese older adults. Meanwhile, although the former study has clarified how SES and DD were associated with HRQoL, respectively, the combined association of them with HRQoL was less known.

To contribute to a deeper understanding of the underlying mechanisms, the aim of the study was to examine the combined association of SES and DD with HRQoL. It also sought to explore whether DD played a mediating role in the relationship between different SES and HRQoL. We hypothesized that higher levels of DD could attenuate the impact of SES on HRQoL in Chinese older adults.

## Materials and methods

### Sample and participants

A questionnaire-based cross-sectional study was undertaken in all 11 cities of Shanxi Province (Taiyuan, Datong, Yangquan, Changzhi, Jincheng, Shuozhou, Jinzhong, Yuncheng, Xinzhou, Linfen, and Lvliang) using a multistage stratified sampling method. First, according to the order of districts (counties) on the government's website, each district (county) in every city was numbered. Second, two (districts) counties in each city were selected using the random number table, and then, two communities (administrative villages) were drawn from each district (county) in the same way. Third, considering the different scales of each community (administrative village), once again, we select 1 to 2 residential communities (natural villages) from each community (administrative village) by the random number table. Finally, we obtained older people's name lists and numbered them in each community or natural village. A random number table was also applied to select older adults who meet the inclusion criteria in this session.

The inclusion criteria for this study were as follows: (1) being aged 60 and above and (2) having clear awareness and barrier-free communication skills. Those who had difficulty communicating were excluded.

Before our formal survey, a pre-study had been conducted to ensure the accuracy, validity, and understandability of the questionnaires. The pre-study was conducted in Taiyuan, Shanxi Province, with 137 questionnaires distributed and 135 returned. The pre-study data were not included in the formal study. Additionally, the results demonstrated that the questionnaire had good reliability and validity and could be well understood by older adults.

All participants were interviewed face-to-face using a structured questionnaire by trained interviewers with medical knowledge in Shanxi Province, China. We used face-to-face interviews instead of self-complete questionnaires because some older adults were illiterate, and some of them could not read or write due to poor vision, hand tremors, or other reasons. The study involved 3,266 older adults, of whom 3,250 completed the questionnaire effectively, with an effective response rate of 99.51%.

### Measurements

#### Socioeconomic status

In our study, occupation before retirement (unemployed, peasant, worker, technician, enterprise, and institution personnel), educational level (illiterate/semi-illiterate, primary education, secondary education, and higher education,) and personal monthly income (<1,000 RMB, 1,000–2,999 RMB, >3,000 RMB) were selected to measure the older adults' SES (6). As a general method to generate the SES index, the principal component analysis (PCA) was employed in this study (28). Then, we classified the SES index by quartiles into four levels: 0 = lowest SES, 1 = lower middle SES, 2 = upper middle SES, and 3 = highest SES.

### Diet diversity

In our study, DD was accessed by diet diversity score. According to the Chinese dietary guidelines for Chinese populations (17), we investigated the frequency of consumption of ten food groups: staple foods (cereals, tubers, and beans), vegetables, fruits, eggs, aquatic products, meat and poultry, soybeans and nuts, milk and dairy products, salt, and oil. We asked the participants how often they ate one of the foods above, and the answers were recorded as “almost every day,” “at least once a week,” “at least once a month,” “not every month,” and “eschew.” For each of the food groups, a score of 1 was given if they answered “almost every day” and no points were given otherwise (29). As salt and oil are essential components of the Chinese daily diet, they were excluded when assessing the DD score (27). In this way, the DD score was accessed by adding up the scores of eight food groups, and the DD score ranged from 1 to 8. A higher DD score indicated a more diverse diet.

### Health-related quality of life

In this study, HRQoL was estimated by the European Quality of Life Five Dimension Five Level Scale Questionnaire (EQ-5D-5L), which was translated into Chinese in 2002 by Luo et al. (30). The EQ-5D instrument is a generic measure of health status with a descriptive system that consists of five dimensions covering mobility (MO), self-care (SC), usual activities (UA), pain/discomfort (PD), and anxiety/depression (AD). Each dimension had five levels of response: 1, no problem; 2, slight problem; 3, moderate problem; 4, severe problem; and 5, extreme problem. As a result, a total of 3,125 unique health status statements can be combined in this system. The EQ-5D utility scores were calculated based on the recently available Chinese value set (31). The score ranges from −0.391 to 1, where 1 represents full health (1,1,1,1,1), and the lowest score is −0.391, which represents a health status worse than death (5,5,5,5,5). The internal consistency measured by Cronbach's alpha was 0.894 in our sample.

### Control variables

We identified potential confounders for HRQoL based on the existing studies (32, 33). Potential confounders included age, gender, residential area, marital status, family size, physical activities, body mass index (BMI), and the number of chronic diseases.

Demographic information was collected by self-report. The family size information was measured based on the question “How many people live with you now (including yourself)?” The answers were grouped as live alone; 2 people; 3–4 people; 5 people; and above. Physical activity level was measured by the International Physical Activity Questionnaire long-form (IPAQ), which consists of 27 questions reflecting on the last 7 days' activities (34). Based on the Chinese guidelines for data processing and analysis concerning the IPAQ (35), participants' physical activity was divided into three levels, including 1 = low, 2 = moderate, and 3 = high. BMI was calculated by weight (kg) divided by squared height (m^2^). According to the Chinese criteria, participants' BMI was classified as underweight (<18.50 kg/m^2^), normal (18.50–23.99 kg/m^2^), overweight (24.00–27.99 kg/m^2^), and obese (≥28.00 kg/m^2^) (36). Information on chronic diseases was collected through self-reporting and supported by diagnostic evidence from the medical records or physicians' prescriptions.

### Statistical analysis

Data were analyzed using SPSS version 24.0 (IBM, Armonk, NY, USA). Categorical variables are presented as the number of participants and compared across SES quartiles by the chi-square tests. Continuous variables are presented as means and standard deviations, compared across SES quartiles by Kruskal–Wallis test because of the non-normal distribution of both the DD score and EQ-5D utility score. The association between the continuous variables was examined using Pearson's correlation.

To examine the combined association of SES and DD on HRQoL, DD scores were grouped into two groups by the mean score of 5 (high: >5, low: ≤ 5). DD groups were combined in pairs with SES divided by quartiles and generated a total of eight combinations as follows: low SES/ low DD; low SES/ high DD; middle low SES/ low DD; middle low SES/ high DD; upper high SES/ low DD; upper high SES/ high DD; high SES/ low DD; and high SES/ high DD. A generalized linear model (GLM) with a Gamma distribution and a log link was applied to investigate the combined association of SES and DD on HRQoL. As the GLM required none-negative values, EQ-5D disutility score (disutility score = 1-utility score) was generated and entered as the dependent variable in this study (37).

A common method bias test was performed using Harman's single factor test to control for common method bias effects. If there is a problem with the common method variance, the first unrotated factor extracted from the factor analysis would account for a large proportion of the total variance. The threshold was 40% according to the former study (38).

The mediation analysis was conducted to establish a mediating model of three variables with adjustment for the above-mentioned control variables by the PROCESS macro developed by Hayes. In the mediation model, SES was determined to be the independent variable (IV), and HRQoL was the dependent variable (DV). DD (MV) was used as the pathway from SES to HRQoL. The total, direct, and indirect effects were estimated by the SPSS PROCESS macro (model 4). A total effect (c) refers to the relationship between the IV and DV without controlling for the MV. A direct effect (c') refers to the relationship between the IV and DV after controlling for the MV (DD) and other control variables. The indirect effects of the mediation analysis were the effects of the IV on the DV through the MV. To test the mediation role of DD between SES and HRQoL, a bootstrap estimation procedure was conducted with 5,000 bootstrap samples. According to the bootstrap test, the effect was significant when the path coefficient of a 95% CI did not overlap 0 (39). All the analyses were performed with SPSS 23.0 at a significance level of 0.05.

## Results

Table 1 shows the sample characteristics by SES quartiles. There were 3,250 participants in total, with an average age of 69.65 ± 6.77 years. Different SES groups showed a statistically significant difference in both the EQ-5D utility and DD scores, as well as other variables including age, gender, residential area, marital status, family size, physical activities, BMI, and the number of chronic diseases (*p* < 0.05).

**Table 1:** Characteristics of the participants across socioeconomic status quartiles (*N* = 3,250).

**Variables**	**Socioeconomic status quartiles** ^**a**^				***P***
	**Lowest**	**Lower middle**	**Upper middle**	**Highest**	
Number of participants	562	1,063	752	873	
**Age group**					
60–70	218	571	473	507	<0.001
71–80	239	412	223	290	
>80	105	80	56	76	
**Gender**					
Male	133	426	443	513	<0.001
Female	429	637	309	360	
**Residential area**					<0.001
Urban	158	214	409	672	
Rural	404	849	343	201	
**Marriage status**					
Married	344	770	625	743	<0.001
Others	218	293	127	130	
**Body mass index (BMI)**					0.019
Underweight	79	147	100	126	
Normal	312	639	459	560	
Overweight	114	196	145	137	
Obesity	57	81	48	50	
**Family size**					
Live alone	15	21	14	14	<0.001
2 people	18	43	68	126	
3–4 people	435	806	536	599	
5 people and above	94	193	134	134	
**Physical activities**					<0.001
Low	164	244	169	146	
Moderate	234	396	305	386	
High	164	423	278	341	
**Number of chronic diseases**					<0.001
0	190	418	351	390	
1	159	316	201	240	
2 and above	213	329	200	243	
Diet diversity score	4.26 ± 1.79	4.68 ± 1.68	5.30 ± 1.74	5.90 ± 1.61	<0.001
EQ-5D utility score	0.80± 0.27	0.85± 0.23	0.89± 0.20	0.92± 0.18	<0.001

^a^Socioeconomic status (SES) was grouped by quartiles from low to high.

Table 2 presents the odds ratios (ORs) for the prevalence of HRQoL across combined categories. The adjusted results showed that in comparison with older adults categorized as high SES/ high DD, most other combinations have significantly higher EQ-5D disutility scores (higher EQ-5D disutility score means better HRQoL): low SES/ low DD (*OR* = 1.65, [95% CI 1.41 to 2.92], *p* < 0.001); low SES/ high DD (*OR* = 1.45, [95% CI 1.17–1.80], *p* = 0.001); middle low SES/ low DD (*OR* = 1.43, [95% CI 1.24–1.65], *p* < 0.001); middle low SES/ high DD (*OR* = 1.23, [95% CI 1.03–1.47], *p* = 0.021); upper high SES/ low DD (*OR* = 1.41, [95% CI 1.21–1.65], *p* < 0.001); and high SES/ low DD (*OR* = 1.30, [95% CI 1.10–1.53], *p* = 0.002), except for the dyad of upper high SES/ high DD (*p* > 0.05).

**Table 2:** Combined association of SES and DD on HRQoL according to the EQ-5D disutility score^a^ (*N* = 3,250).

	***OR***	**95% CI**	**^b^ *P***
Low SES/ Low DD	1.65	1.41, 1.92	<0.001
Low SES/ High DD	1.45	1.17, 1.80	0.001
Middle low SES/ Low DD	1.43	1.24, 1.65	<0.001
Middle low SES/ High DD	1.23	1.03, 1.47	0.021
Upper high SES/ Low DD	1.41	1.21, 1.65	<0.001
Upper high SES/ High DD	0.95	0.81, 1.13	0.575
High SES/ Low DD	1.30	1.10, 1.53	0.002
High SES/ High DD	1.00		

SES, Socioeconomic Status; HRQoL, Health Related Quality of Life; DD, Diet Diversity.

^a^EQ-5D disutility score: converted from EQ-5D utility score (disutility score = 1-utility score), higher EQ-5D disutility score represents lower HRQoL; DD scores were grouped into two groups by the mean score of 5 (high: >5, low: ≤ 5).

^b^The analysis was adjusted by age, gender, residential area, marital status, family size, physical activities, body mass index, and the number of chronic diseases.

In terms of the correlation between key variables, prominently, DD score and HRQoL were positively correlated with SES, respectively (for DD, *r* = 0.333, *p* < 0.01; for HRQoL, *r* = 0.182, *p* < 0.01). HRQoL was also significantly and positively correlated with DD (*r* = 0.239, *p* < 0.01). In addition, the results of the common method biases showed that the eigenvalues of seven factors were greater than one in the unrotated factors, implying that the data are explained by more than one factor. In addition, the first factor explained 28.14% of the variance, less than the threshold of 40%. As a result, the problem of common method bias in this study is not serious.

**Figure 1:**
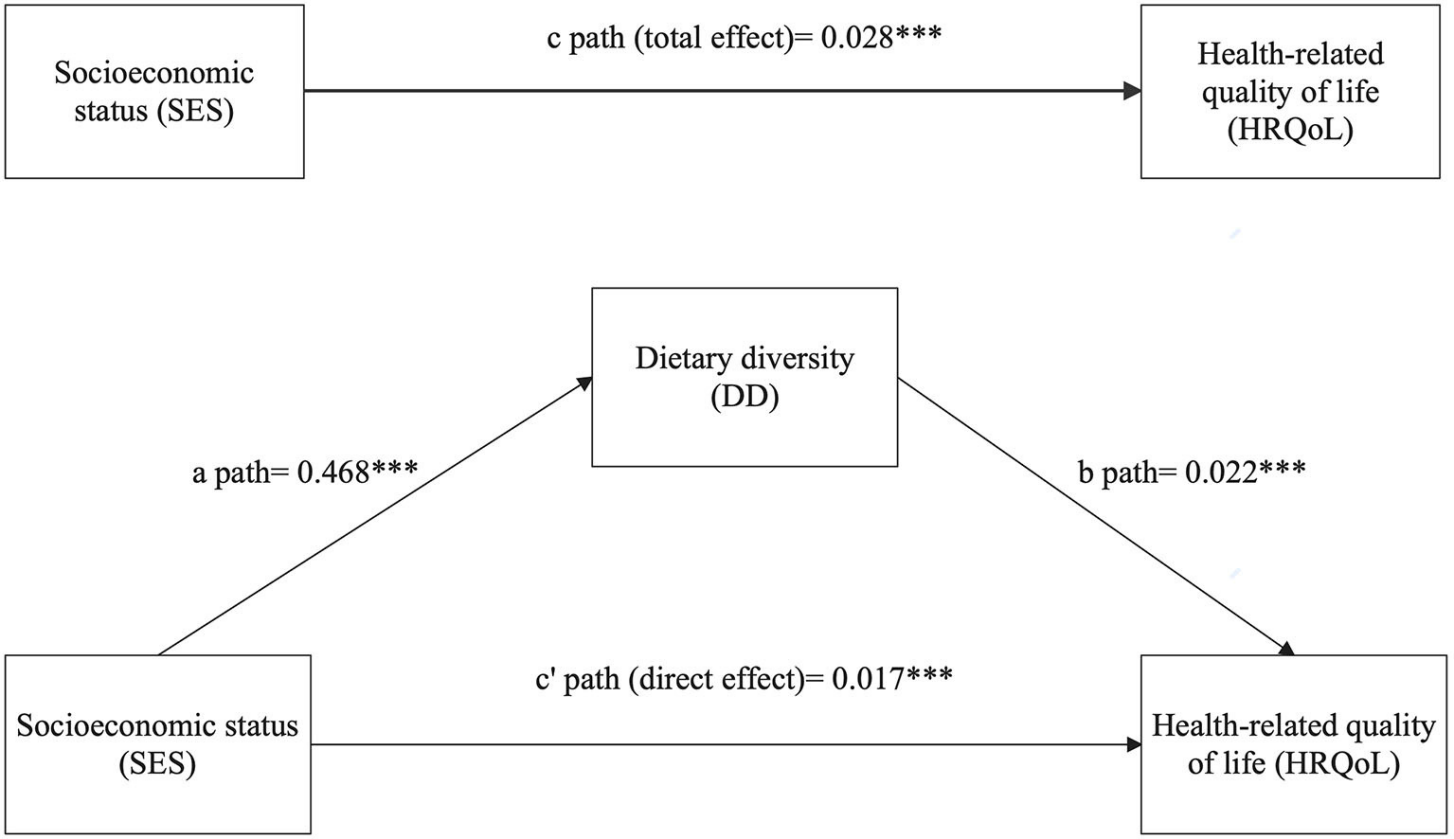
The mediation model of the association between SES and HRQoL through dietary diversity, adjusted for age, gender, residential area, marital status, family size, physical activities, body mass index, and the number of chronic diseases. Path coefficients are shown. ***p < 0.001.

This mediation analysis provided the estimates of path coefficients and significance tests for specified mediational models, as well as the estimates of indirect effects. All paths for the adjusted model are presented in Figure 1. The total effect of SES on HRQoL was path c = 0.028 (*p* < 0.001). The significance of the direct effect of SES on HRQoL (path c' = 0.017, *p* < 0.001) remained significant when the mediator of DD was included in the model, indicating that the mediating effect was partial. Moreover, the total indirect effects through the pathway of SES → DD → HRQoL were 0.011, which accounted for 39.29% of the total effect.

The regression coefficients in the mediation analysis between SES on HRQoL with DD as a mediator are presented in Table 3. The effects of SES on HRQoL were partly mediated by DD. After controlling covaries, the direct effect of SES on HRQoL (*B* = 0.028, *p* < 0.001) remained significant when the mediator of DD was included in the model. In addition, SES was significantly associated with DD (*B* = 0.468, *p* < 0.001), with DD also significantly associated with HRQoL (*B* = 0.022, *p* < 0.001).

**Table 3:** Regression coefficients in the mediation analysis between SES on HRQoL with DD as mediator (*N* = 3,250).

**Criterion**	**Predictors**	***R^2^***	***F***	***B***	***SE***	***t***	**95% CI**	***P***
HRQoL	SES	0.178	77.726	0.028	0.004	6.994	(0.020, 0.035)	<0.001
DD	SES	0.140	58.650	0.468	0.032	14.444	(0.404, 0.532)	<0.001
HRQoL	SES	0.204	83.165	0.017	0.004	4.325	(0.009, 0.025)	<0.001
	DD			0.022	0.002	10.432	(0.017, 0.026)	<0.001

HRQoL, Health Related Quality of Life; SES, Socioeconomic Status; DD, Diet Diversity; age, gender, residential area, marital status, family size, physical activities, body mass index (BMI), and the number of chronic diseases were analyzed as control variables.

## Discussion

In the present cross-sectional study, we explored the combined association of SES and DD with HRQoL among older adults, finding people who have lower SES and DD have higher odds of lower HRQoL. Through the bootstrapping analysis, the current study also confirmed that DD mediated the association between SES and HRQoL, which is in accordance with our theoretical assumption. As DD explained more than one-third of this relationship, the results may further suggest the value and importance of promoting DD for attenuating the negative impact of SES and HRQoL.

The positive association of SES with HRQoL in the current study was consistent with the social causation theory and earlier studies (8, 9, 40). The impact of SES highlighted the importance of graded socioeconomic differences in HRQoL. A recent study in China regarded SES as the main contributor to health inequality measured by the EQ-5D (41). Previous studies also discovered that the indicators of SES including education level, monthly income, and occupation were independently associated with HRQoL. In older Brazilian community-dwelling adults, participants with 5 or more years of education tended to report better HRQoL (42). Household income was identified as a risk factor exacerbating the disparities in the prevalence of poor quality of life in a Chinese longitude study from 1998 to 2018 (22). The findings from Chen et al. show that older adults' previous occupations played a role in affecting their HRQoL in mainland China (43). Low SES older adults have dual vulnerabilities and their HRQoL demands more government and academic attention, given the current pro-rich inequality in health status among Chinese older adults (44).

Our findings suggest that SES had a major influence on participants' DD echoed with a recent study in China that higher SES, including more education, family income, and perceived income status, was associated with higher DD (45). Another study emphasized the role of material resources, showing that income has an impact on diet: the higher the income, the more diverse the diet (46). Income reflects a person's purchasing power and is a good indicator of a person's economic resources. When people are making their dietary-related consumption decisions, income levels could limit their budgets. The tighter the budget is, the more limited choices they face, which would hinder the DD practice. However, money is not everything. Fraval et al. found that increasing income did not necessarily lead to improved DD in a study in Sub-Sahara Africa (47). Other social resources such as educational level were also found to be associated with DD (48). Education is a form of human capital, and people with high levels of education are more likely to have stable careers and income, which enable them to support investments in health including having a diverse diet. Nutrition awareness may be another issue as a previous study pointed out that among older adults, less educated people have lower nutrition awareness (49), and poor nutrition awareness was found to be associated with lower HRQoL (50). Put together, stronger purchasing power, higher willingness to stay healthy, and better nutrition awareness that came with more advanced SES may further help to explain people's tendency to consume a diverse diet.

According to our findings, maintaining a diverse diet was positively associated with better HRQoL, which was in line with the former study in China that a higher DD score was associated with better health, such as being less likely to experience higher-level psychological stress (51), having reduced risk of bladder cancer (52) and a lower risk of frailty (53). DD had the potential to promote HRQoL through the pathway of nutrition intakes (54, 55). Increased DD was associated with better multi-nutrient intake such as calories, protein, nucleic acid, vitamin C, and zinc, as compared to the individuals with lower DD (56). A diverse diet with adequate protein intake could boost the anabolic activity of skeletal muscle and stimulate muscle protein synthesis (57), preventing the negative impact of age-related degradation in skeletal muscle mass and function on older adults' HRQoL (58). Additionally, as shown in a local study among Chinese centenarians, higher DD was also a protective factor against mental disorders (29).

The findings of the present study suggest that in comparison with the individuals with both high SES and high DD, another relatively low SES either in combination with high or low DD was found to be associated with decreased HRQoL except for the combination of upper high SES and high DD. Although sub-average SES was significantly associated with higher odds of lower HRQoL, the protective role of high DD was still evident in ameliorating the negative impact of relatively low SES. It is worth noting that even for older adults with more advanced SES, lower DD was also associated with decreased HRQoL. Considering the fact that when people get older, they are more likely to consume a monotonous diet (59), and it was valuable to pay attention to older adults' DD for ensuring their quality of late life. In terms of the exception for older adults who have high DD, no significant difference in HRQoL between those from upper high and high SES groups was observed in our study. The finding suggested that the HRQoL could be more secure for older adults having both above-average SES and DD. The study revealed that DD could play a mediating role in bridging the HRQoL inequity caused by the SES gap, slightly weakening this relationship and providing a new perspective explaining the relationship between SES and HRQoL. Our findings further indicated the priority targets for future DD interventions, as the research had suggested that the cumulative health disadvantage caused by SES could be ameliorated by lifelong health-related behavior (60). DD screening and assessment have the practical merit of being fast, widely applicable, and easy to understand for older people (13, 29), and increasing DD could be one promising intervention strategy to promote HRQoL in later life.

## Limitations

There are some limitations to this study that should be addressed. First, given the cross-sectional design of the present study, it is not possible to make causal inferences. Second, participants' consumption of food groups was collected through only self-reported, which may exist recall bias as well as social desirability bias. There was also unintentional bias as the participants who have difficulties in communication were excluded from our research without having proxy interviews. Third, the study participants were recruited locally from Shanxi Province, which does not represent other areas in China.

## Conclusion

Based on the study of 3,250 older adults in Shanxi province, China, the results not only presented the combined association of SES and DD on HRQoL but also suggested that the association between SES and HRQoL was partly mediated by DD. The findings had some implications for HRQoL enhancement as it is crucial to ensure that older populations from various socioeconomic backgrounds could equally enjoy their quality twilight years. Given the findings that higher DD could attenuate the negative impact of SES and HRQoL in Chinese older adults, village clinics and community health stations should work together on intervention programs encouraging older adults to adopt a diverse diet for improving or maintaining HRQoL.

## Data availability statement

The original contributions presented in the study are included in the article/supplementary material, further inquiries can be directed to the corresponding author.

## Ethics statement

The studies involving human participants were reviewed and approved by Shanxi Medical University. The patients/participants provided their written informed consent to participate in this study. Written informed consent was obtained from the individual(s) for the publication of any potentially identifiable images or data included in this article.

## Author contributions

CZ designed this study and served as the lead writer. JZ did the data interpretation and drafted the manuscript. SX and LS were involved in the study design and gave many valuable comments on the draft. YX and XZ participated in data analysis and critically revised the article. YK and XB helped with the language and format editing of this manuscript. XL, YC, YL, and GZ participated in data collection and post-works. All authors have read and approved the manuscript.
